# Impact of Vitamin C on Gene Expression Profile of Inflammatory and Anti-Inflammatory Cytokines in the Male Partners of Couples with Recurrent Pregnancy Loss

**DOI:** 10.1155/2022/1222533

**Published:** 2022-03-22

**Authors:** Farzaneh Fesahat, Efat Norouzi, Seyed Mohammad Seifati, Saeideh Hamidian, Akram Hosseini, Fateme Zare

**Affiliations:** ^1^Reproductive Immunology Research Center, Shahid Sadoughi University of Medical Sciences, Yazd, Iran; ^2^Departments of Anatomical Sciences, Isfahan University of Medical Sciences, Isfahan, Iran

## Abstract

Immune system disorders and increased inflammation in the male reproductive system can lead to fetal risk in the early stages of development and implantation. Antioxidants such as vitamin C can play a protective role against sperm inflammatory reactions. This study aimed to evaluate the effect of vitamin C on the expression of inflammatory and anti-inflammatory cytokine genes in the male partners of couples with recurrent pregnancy loss. In this randomized clinical trial, twenty male partners of couples with RPL were examined for sperm parameters and expression profile of some inflammatory and anti-inflammatory cytokine genes before and after treatment with vitamin C. There was a statistically significant higher rate of normal morphology and sperm concentration in each patient before and after treatment with vitamin C (*p* ≤ 0.05). The mRNA levels of interleukin 6 and tumor necrosis factor-alpha were significantly decreased in the sperm of patients after treatment with vitamin C compared to before treatment. In contrast, the gene expression levels of interleukin 4 and transforming growth factor-beta showed a significant increase in the sperm of patients after treatment with vitamin C. Oral daily administration of vitamin C may be effective in the fertility potential of male partners of couples with RPL not only through the improvement of the sperm parameters but also by modulating the expression profile of inflammatory and anti-inflammatory genes. Further studies on protein levels are needed to clarify the role of TNF-*⍺* and IFN-*γ* as a prognostic value in evaluating the recurrent abortion risk in infertile male partners. This trial is registered with IRCT20180312039059N1.

## 1. Introduction

According to the American Society of Reproductive Medicine, recurrent pregnancy loss (RPL) is two or more consecutive miscarriages before 20–24 weeks of gestation [1]. Although distinct risk factors for RPL including genetic factors, immune dysfunction, and autoimmune disorders, hormonal factors, uterine anatomy, infections, and thrombosis have been reported, the etiology of over 50% of RPLs is yet unknown [2]. Recent studies suggest that male partners of couples with RPL who have a normal karyotype may have a high percentage of sperm abnormalities. Male gamete with genetic abnormalities or epigenetic alterations may fertilize the oocyte and severely affect early embryonic development [3, 4]. Recent studies showed that abnormal numbers or rearrangements of chromosomes in the male parent lead to higher abortions, reduced fertility, or infertility [5, 6]. Sperm parameters may be damaged through aging or effects of drug, immunological, radiation, and environmental factors such as life style, diet quality, physical activity, and infections. One study declared that increased sperm abnormalities or aneuploidy may increase the risk of miscarriage [7].

Furthermore, immunological factors may be involved in sperm abnormalities in male partners of couples with RPL [8]. Evidence suggests that physiological exposure to semen has modulatory effects on the immune system. Seminal plasma includes immunosuppressive factors such as TGF-*β*, IL-10, and prostaglandin *E* (PGE) [9]. Semen plasma contains PG and polyamine along with TGF-*β*, which recruits inflammatory cells in the uterus that suppresses the immune system. Based on this study, TGF-*β* and activin in seminal plasma may affect the function of cervical immune cells after coitus [10]. Taima et al. showed that the production of inflammatory cytokines by endometrial NK cells is regulated by seminal plasma exposure, indicating immune compatibility between semen and endometrium [11]. Seminal plasma destroys free radicals with antioxidant factors such as vitamin C, carnitine, tyrosine, uric acid, glutathione peroxidase, and pyridoxine [12].

Vitamin C (ascorbic acid) is part of the human diet with various physiological functions that its deficiency is associated with many symptoms such as malaise, fatigue, loss of appetite, petechiae bleeding, purpura, swollen or bleeding gums, corkscrew hairs, and follicular hyperkeratosis [13–15]. Vitamin C may play an essential role in the testicular antioxidant defense system and support spermatogenesis [16]. In addition, it has potent anti-inflammatory properties. It has been reported that vitamin C could regulate the inflammatory status by decreasing IL-6 and hs-CRP in obese patients with hypertension and/or diabetes [17].

Male fertility disorders at heavy costs are a global issue, and many studies have been conducted on the effect of antioxidants on mammalian sperm. Therefore, the present study aimed to evaluate the effect of vitamin C on the gene expression of inflammatory and anti-inflammatory cytokine as well as sperm parameters in the male partners of couples with RPL.

## 2. Materials and Methods

### 2.1. Participants

This randomized clinical trial was designed as an alternative investigation of our previous study that was registered in the Iranian Registry of Clinical with the national ID no. IRCT20180312039059N1 [18]. Twenty male partners of infertile couples with RPL due to male factor infertility were randomly selected among who referred to Yazd Research and Clinical Center for infertility using a random number table. Informed consent was obtained from all participants. In addition to medical records, each patient was asked to complete a questionnaire based on demographic information and inclusion and exclusion criteria. Regarding the exclusion criteria, the questions were adjusted based on consumption or nonconsumption of chemical substances so far. The inclusion criteria of participants were having a history of two or more than RPL, age less than 40 years, sperm concentration of 7–14 million per ml, total sperm motility <40%, and sperm with normal morphology <4% according to the 2010 World Health Organization (WHO) criteria [21]. The men with a history of alcohol use, any consumption of tobacco, antidepressants, and antioxidants, or having obesity (based on body mass index), diabetes, and varicocele disorders were excluded from this study. Also, exclusion criteria were considered for the female partners, such as hormonal imbalance, chromosomal changes, tubal obstruction, and bacterial or viral infections. Each semen samples were evaluated before and after treatment with vitamin C. Briefly, participants were prescribed 250 mg of vitamin C daily in tablet form (Avicenna Company, Tehran, Iran) by a urologist for 3 months [18]. The individuals were recommended to consume fruits and vegetables through the intervention period and not to drink soft drinks, soybeans, canned foods, and even unnecessary use of mobile phones or laptops. Before and after treatment with vitamin C, the sperm parameters and gene expression levels of inflammatory and anti-inflammatory cytokines were assessed. The study was conducted by an experienced laboratory technician who was blind to the allocation of participants. All procedures performed in this study were following the ethical standards of the institutional or national research committee and the 1964 Helsinki declaration and its later amendments or comparable ethical standards.

### 2.2. Collection and Analysis of Semen

Semen samples were collected by masturbation after at least 2-3 days of abstinence. The samples were liquefied at 37°C for at least 30 min before semen analysis according to the WHO 2010. The concentration and the motility of the samples were determined under light microscopy at ×400 magnification. The Diff-Quik staining method was used to the assessment of sperm morphology.

### 2.3. Cytokine Genes Expression

In this study, relative gene expression methodology including RNA extractions, quality control of extracted RNA, cDNA synthesis, and relative gene expression assessments were performed according to our previous studies [18, 20]. Total RNA molecules were extracted from all sperm samples using a total RNA extraction kit (Parstous Biotechnology, Iran), according to the manufacturer's instructions. RNA concentration and quality were evaluated using the spectrophotometer with absorbance at 260 nm (Photobiometer, Eppendorf, Germany). cDNA synthesis of each RNA (100 ng/µL) was performed using the RevertAid First Strand cDNA Synthesis Kit according to the manufacturer's protocol (Parstous Biotechnology, Iran). Specific primers for IL-10, IL-4, IL-6, TNF-*⍺*, IFN-*γ*, TGF-*β*, and GAPDH (reference gene) were used for real-time quantitative reverse transcriptase-polymerase chain reaction (qRT-PCR) (Table 1). qRT-PCR was performed by SYBR Green Master Mix (Amplicon) by using the StepOne system (Applied Biosystems, CA, USA). For each reaction, cDNA (2 *μ*L), forward primer (1 *μ*L), reverse primer (1 *μ*L), the master mix (10 *μ*L), and 6 *μ*L nuclease-free water were adjusted to a total of 20 *μ*L. All the reactions were carried out in duplicate. The qRT-PCR protocol composed of the following: 10 min at 95°C, followed by 40 cycles of amplification stage at 95°C for 15 s, 60°C for 30 s, and 72°C for 30 s. A melting curve stage was run after the cycling stage. The analysis of the data for relative gene expression was conducted by the 2^−△△CT^ method.

### 2.4. Outcome Measurement

Clinical pregnancy was confirmed by detection of gestational sac with a fetal heartbeat. Abortion rate was defined as clinical pregnancy loss before 20^th^ week of gestation from pregnancies. A live birth was recorded when a fetus exits the maternal body and immediately shows vital sign.

### 2.5. Statistical Analysis

All statistical analyses were performed using the SPSS software for Windows ver. 20.0 (IBM Corp., Armonk, NY, USA). Data were reported as means ± standard error of the mean. The paired *t*-test was used to analyze the data before and after treatment with vitamin C. *P* less than 0.05 was considered a significant value.

## 3. Results

The results showed no significant difference in the sperm concentration before and after treatment (76.95 ± 3.76 vs. 86.10 *±* 3.73, *p* ≥ 0.05). In contrast, the morphology of sperm was significantly improved after treatment when compared with before vitamin treatment (2.44 ± 0.29 vs. 6.23 ± 0.56, *p* < 0.001). Also, the total motility of sperm showed a significantly higher rate following the treatment with vitamin (*p* < 0.001). The clinical pregnancy, live birth, and abortion rates were 14/20 (70%), 8/20 (40%), and 4/20 (20%), respectively.

Regarding the gene expression levels of IL-4 and TGF-*β*, a significantly higher mRNA level was seen between the samples before and after treatment (*P*=0.01 and *P*=0.02, respectively). However, the relative expression of IL-6 and TNF-*α* were significantly decreased in the sperm of patients after treatment compared to before treatment. IFN-*γ* was significantly upregulated after treatment (*P*=0.002) (Table 2). A significant reverse correlation was seen between recurrent abortion and TNF-*⍺* and IFN-*γ* genes expression in sperm cells of infertile men before treatment (*r* = −0.41, *r* = −0.39 and *p*=0.004, *p*=0.01, respectively).

## 4. Discussion

Cytokines play an important role in the human reproductive process by modulating the immune system [21]. Cytokines may be involved in all stages of reproduction and affect pregnancy outcomes [22]. Cytokines such as IL-4, IL-6, and IL-10 seem to cause successful pregnancies, while cytokines such as TNF-*α* and interferon (IFN)-*γ* are harmful to pregnancy and prevent fetal growth and development [23]. Daher et al. investigated the serum levels of Th1 and Th2 cytokines in peripheral blood of 29 women with RPL compared with 27 healthy women as controls. They showed that the levels of IFN-*γ* and TNF-*α* were higher in women with the RPL group compared to the controls. However, IL-6 and TGF-*β* were not significantly different between the two groups. They concluded that Th1 cytokines may play an essential role in the pathogenesis of RPL [24].

The present study aimed to evaluate the effect of vitamin C on the expression of inflammatory and anti-inflammatory cytokine genes in the male partners of couples with recurrent pregnancy loss. Vitamin C led to decrease proinflammatory cytokines gene expression (IL-6 and TNF-*α*) and increase anti-inflammatory cytokines gene expression (IL-4 and TGF-*β*). However, vitamin C consumption may not affect the gene expression of IL-10. In the present study, IFN-*γ* gene expression was significantly increased. Molina and colleagues conducted an in vitro study that aimed to evaluate the effect of vitamin C on functional parameters of healthy human lymphocytes. The findings showed a significant reduction in IFN-*γ* secretion and a significant increase in IL-10 levels of lymphocyte cells after treatment with 100 *μ*M vitamin C. Aforementioned results were in contrast with our findings that are properly due to the fundamental differences in methodology and study design of two studies [25]. Sanka et al. investigated a set of cytokine levels in seminal plasma in infertile men with or without genital tract inflammation. They showed that proinflammatory cytokines modulated the oxidative stress and led to reasonable improvements in sperm parameters of infertile men [26]. Studies demonstrated that the immune system function in male infertility plays an effective role in the pathogenesis of sperm cells. For instance, cytokines secreted by various cells of the male reproductive system such as Sertoli cells and Leydig cells are involved in male fertility and may affect steroidogenesis, spermatogenesis, and sperm function [27, 28]. A high level of the proinflammatory cytokines, such as IL-1*β*, IL-6, and TNF-*α*, causes a decrease in sperm quality by inducing oxidative stress and lipid peroxidation [29]. Chyra-Jach et al. showed that infertility and sperm abnormalities in males with asthenospermia and oligoasthenospermia may be promoted through decreased antioxidant activity, increased cytokine levels, and proinflammatory chemokines in semen [30]. Although Chyra-Jach investigated cytokines at the protein level, the results of this study are consistent with our data, performed at the gene level.

The effect of vitamin C on sperm parameters has been reported in several studies [18, 31]. In line with our previous study [18], the results of the present research showed that vitamin C supplementation could significantly improve the motility and morphology of sperm in male partners of infertile couples with RPL. Moreover, previous results showed that the pregnancy rate was increased in women with RPL after administration of vitamin C by associated male partners [18]. Regarding our findings, the modulatory effect of vitamin C on the immune system potentially could be one of the main causes of the improving pregnancy rates in these patients. Nazari et al. investigated the effect of vitamin E and zinc on the sperm parameters of 60 couples with RPL. Consistent with the present study, they showed that sperm motility and morphology improved significantly after antioxidants use [32]. Akmal et al. explained that consumption of vitamin C by infertile men may improve the count, motility, and morphology of sperm and may increase pregnancy [31]. Taken together, it seems that vitamin C supplementation may remarkably improve the quality of semen along with the pregnancy rate in men with male factor infertility and a history of RPL.

To our knowledge, there is little available evidence-based information about the role of cytokines in recurrent miscarriage, since more clinical trials still require before one supplement could be administered as treatment options for infertile couples with RPL. Also, little information is available about the exact mechanism regarding the relationship between the aberrant expression of the inflammatory/anti-inflammatory cytokines in spermatozoa and RPL. However, according to the literature, Sertoli, Leydig cells, and leukocytes residing in the testis, such as monocytes, macrophages, mast cells, secrete a variety of cytokines that balance inflammatory and anti-inflammatory cytokines [33]. Cytokines play an important role in gap junctions and severely affect spermatogenesis. If the balance between inflammatory and anti-inflammatory cytokines in the testis is disturbed, which leads to an increase in the level of inflammatory cytokines, it could lead to an increase in reactive oxygen species (ROS) in the testicular environment and creating transient openings to pass the spermatocytes following the disruption of the Sertoli cell cytoskeleton [34, 35]. In this case, the abnormal cells may get out of transient routs accompanied with the adult cells. In addition, the definitive role of ROS in DNA damage should not be overlooked [18, 36]. If abnormal sperms are fertilized with female gametes, they may interfere with the embryo implantation process and cause RPL, which may be one of the reasons for RPLs of unknown cause. It is also recommended more clinical trials with a larger sample size along with control individuals, evaluation of cytokines in different levels (serum, seminal plasma), and the use of semen of fertile men as positive control are recommended in future studies.

## 5. Conclusion

Oral daily administration of vitamin C may be effective in the fertility potential of male partners of couples with RPL not only through the improvement of the sperm parameters but also by modulating the expression profile of inflammatory and anti-inflammatory genes. Further studies on protein levels are needed to clarify the role of TNF-*⍺* and IFN-*γ* as a prognostic value in evaluating the recurrent abortion risk in infertile male partners.

## Figures and Tables

**Table 1:** Oligonucleotide primers.

Accession numbers	Gene	Primer sequence (5′-3′)	PCR product (bp)
NM_001382624.1	IL-10	F: GGTTGCCAAGCCTTGTCTGA R: AGGGAGTTCACATGCGCCT	101
NM_001354990.2	IL-4	F: ACCTCGACTCGCCTACAAAG R: TTCCTGTCGAGCCGTTTC	184
NM_000660.7	TGF-*β*	F: GTACCTGAACCCGTGTTGCT R: GAACCCGTTGATGTCCACTT	288
NM_001371096.1	IL-6	F: GGTACATCCTCGACGGCATCT R: GTGCCTCTTTGCTGCTTTCAC	81
NM_000594.4	TNF-*⍺*	F: CCCAGGCAGTCAGATCATCTT R: AGCTGCCCCTCAGCTTGA	85
NM_000619.3	IFN-*γ*	F: AGCGGATAATGGAACTCTTTTCTT R: AAGTTTGAAGTAAAAGGAGACAATTTGG	103
NM_001357943.2	GAPDH	F: GTATGACAACGAATTTGGCTACAG R: GTCTCTCTCTTCCTCTTGTGCTCT	119

IL-10, interleukin 10; IL-4, interleukin 4, TGF-*β*, transforming growth factor-beta; IL-6, interleukin 6; TNF-*α*, tumor necrosis factor-alpha; IFN-*γ*, interferon-gamma; GAPDH, glyceraldehyde 3-phosphate dehydrogenase; F, forward; R, reverse.

**Table 2:** Comparison of relative cytokines genes expression before and after treatment with vitamin C.

Genes	Before treatment (*N* = 20)	After treatment (*N* = 20)	*P* value
IL-10	0.95 ± 0.21	0.49 ± 0.08	0.13
IL-4	0.016 ± 0.016	0.28 ± 0.08	0.01^*∗*^
TGF-*β*	0.03 ± 0.02	0.63 ± 0.31	0.02^*∗*^
IL-6	2.01 ± 0.39	1.16 ± 0.04	<0.001^*∗*^
TNF-*⍺*	0.16 ± 0.15	0.11 ± 0.04	0.007^*∗*^
IFN-*γ*	0.48 ± 0.25	2.21 ± 0.38	0.002^*∗*^

Data are presented as the mean ± SEM according to the Wilcoxon test. ^*∗*^*P* < 0.05 was considered as a significant value. IL-10, interleukin 10; IL-4, interleukin 4;TGF-*β*, transforming growth factor-beta; IL-6, interleukin 6; TNF-*⍺*, tumor necrosis factor-alpha; IFN-*γ*, interferon-gamma.

## Data Availability

The data used to support the findings of this study are included within the article and are available from the corresponding author upon request.
